# Assessing the effects of a drought experiment on the reproductive phenology and ecophysiology of a wet tropical rainforest community

**DOI:** 10.1093/conphys/coad064

**Published:** 2023-09-19

**Authors:** Nara Vogado, Susan G Laurance, Michael J Liddell, Jayden E Engert, Christopher M Wurster, Michele Schiffer, Andrew Thompson, Cassandra Nichols, Lucas A Cernusak

**Affiliations:** Centre for Tropical Environmental and Sustainability Science, College of Science and Engineering, James Cook University, 1/14-88 McGregor Rd Smithfield, Cairns, 4870, Australia; Centre for Tropical Environmental and Sustainability Science, College of Science and Engineering, James Cook University, 1/14-88 McGregor Rd Smithfield, Cairns, 4870, Australia; Centre for Tropical Environmental and Sustainability Science, College of Science and Engineering, James Cook University, 1/14-88 McGregor Rd Smithfield, Cairns, 4870, Australia; Centre for Tropical Environmental and Sustainability Science, College of Science and Engineering, James Cook University, 1/14-88 McGregor Rd Smithfield, Cairns, 4870, Australia; ARC Centre of Excellence for Australian Biodiversity and Heritage, James Cook University, 1/14-88 McGregor Rd Smithfield, Cairns, 4870, Australia; Daintree Research Observatory, James Cook University, Cape Tribulation, 4873, Australia; Daintree Research Observatory, James Cook University, Cape Tribulation, 4873, Australia; La Trobe University, Yarraville, Victoria, Australia; Centre for Tropical Environmental and Sustainability Science, College of Science and Engineering, James Cook University, 1/14-88 McGregor Rd Smithfield, Cairns, 4870, Australia

**Keywords:** Climate change, drought, flowering, fruiting, stable isotopes, water use efficiency, Wet Tropics

## Abstract

Climate change is expected to increase the intensity and occurrence of drought in tropical regions, potentially affecting the phenology and physiology of tree species. Phenological activity may respond to a drying and warming environment by advancing reproductive timing and/or diminishing the production of flowers and fruits. These changes have the potential to disrupt important ecological processes, with potentially wide-ranging effects on tropical forest function. Here, we analysed the monthly flowering and fruiting phenology of a tree community (337 individuals from 30 species) over 7 years in a lowland tropical rainforest in northeastern Australia and its response to a throughfall exclusion drought experiment (TFE) that was carried out from 2016 to 2018 (3 years), excluding approximately 30% of rainfall. We further examined the ecophysiological effects of the TFE on the elemental (C:N) and stable isotope (*δ*^13^C and *δ*^15^N) composition of leaves, and on the stable isotope composition (*δ*^13^C and *δ*^18^O) of stem wood of four tree species. At the community level, there was no detectable effect of the TFE on flowering activity overall, but there was a significant effect recorded on fruiting and varying responses from the selected species. The reproductive phenology and physiology of the four species examined in detail were largely resistant to impacts of the TFE treatment. One canopy species in the TFE significantly increased in fruiting and flowering activity, whereas one understory species decreased significantly in both. There was a significant interaction between the TFE treatment and season on leaf C:N for two species. Stable isotope responses were also variable among species, indicating species-specific responses to the TFE. Thus, we did not observe consistent patterns in physiological and phenological changes in the tree community within the 3 years of TFE treatment examined in this study.

## Teaser

In this study we assessed the effects of an experimental drought on the reproductive phenology and ecophysiology of a rainforest tree community in the Wet Tropics of Queensland. We found that, although the drought increased fruiting activity in the community relative to the reference plot, species-level response was varied in both phenology and physiology.

## Introduction

Reproductive phenological patterns are important ecological processes that influence resource provision for fauna species and ecosystem composition and function. Better understanding the physiological processes that may influence phenological patterns of evergreen tropical rainforests, both at community and population levels, has become essential to better predict tree responses to a changing environment (Butt *et al.,* 2015). While the importance of temperature and water availability in the context of global change biology is well understood, species-level effects are rarely studied in tropical rainforests, with most experimental studies carried out in temperate systems (Ogaya and Peñuelas, 2007). Studies analysing the effect of drought on the phenology of tropical rainforest tree species are rare (but see Brando *et al.,* 2006, Meir *et al.,* 2018), and studies combining physiological proxies with assessments of phenological responses to environmental changes in tropical rainforests are even rarer (Yoneyama and Ichie, 2019; Vogado *et al.,* 2020). As a result, there is a significant gap in our understanding of how tropical rainforests are responding to changes in climate and how they will respond in the future.

Phenology and physiology of tree species are affected by climate and may be influenced by predicted increases in the intensity and frequency of droughts. In the tropics, flowering and fruiting activities have been found to be affected by climate variables, such as rainfall (Dunham *et al.,* 2018) and temperature (Pau *et al.,* 2013). Photoperiod and solar irradiation (van Schaik *et al.,* 1993; Chapman *et al.,* 2018; Wright and Calderón, 2018) may also be important triggers of variation in tropical plant phenology, although roles of individual drivers are often difficult to disentangle. In humid tropical forests, photoperiod has been suggested to be important for the timing and synchronicity of plant phenology as equatorial regions experience lower seasonality and more constant water availability (van Schaik *et al.,* 1993; Morellato *et al.,* 2000; Borchert *et al.,* 2005; Calle *et al.,* 2010; Borchert *et al.,* 2015). Additionally, temperature and water availability are important for numerous physiological processes in tropical tree species and, through changes in resource availability, may have an important indirect influence on phenological processes. This combined effect of temperature and water availability on phenological patterns and physiological processes of tropical tree species has important consequences for the structure and function of tropical forests, the persistence of tropical tree species and potentially the persistence of rainforests more broadly.

Trees are expected to change their reproductive activities as a result of physiological responses to drought. For example, non-structural carbohydrate (NSC) synthesis may be impacted by limitation on photosynthesis by stomatal closure brought on by drought conditions (Nepstad *et al.,* 2002; Brando *et al.,* 2006). Species that are more capable of acclimating to drought might exhibit a higher water use efficiency (WUE), allowing them to maintain uptake of carbon for photosynthesis while reducing water loss through transpiration and avoiding embolisms (McDowell *et al.,* 2008). However, in a throughfall experiment in the Amazon rainforest (Metcalfe *et al.*, 2010), trees have been found to shift allocation of NSC, which may enable trees under drought stress to maintain production of flowers and fruits. On the other hand, a lower capacity to increase WUE or make more effective use of water through rooting depth adjustments might cause a reduction in flower and fruit production as the plants are placed under both water and carbohydrate stress.

The use of stable isotopes, as an integrative measure of physiological processes, provides a useful tool to understand how trees are responding to climate change (Snyder *et al.,* 2022), in particular through the relationship between intrinsic WUE (WUEi; equal to the ratio of photosynthesis to stomatal conductance) and *δ*^13^C values (Cernusak *et al.,* 2013). WUEi can be assessed using the stable carbon isotope composition of plant tissue, represented by the *δ*^13^C value (Cernusak, 2020), since the process of photosynthesis is impacted by stomatal activity, that is by balancing water loss with carbon gain. Here, we hypothesized that the throughfall exclusion drought experiment (TFE) would result in an increase in *δ*^13^C values due to an increase in WUE, and that species that do not acclimate would maintain or decrease their WUEi. Additionally, the *δ*^18^O value of plant tissue may be used as a tool to understand plant water relations, especially transpiration rates and water sources (Barbour, 2007). Species under drought conditions would be expected to have higher *δ*^18^O values due to a decrease in transpiration rates as a result of more closed stomata. The nitrogen-stable isotope composition (*δ*^15^N value) of leaf tissue can provide insight into the soil depth at which nitrogen is being acquired, as soil *δ*^15^N generally increases with increasing depth in the soil profile (Craine *et al.,* 2015; Nel *et al.,* 2018; Lorenz *et al.,* 2020). If overall nitrogen availability decreases in response to prolonged drought, due to slower litter decomposition and reduced mineralization in the upper soil layers, an increase in the C:N ratio of new foliage might also be expected (Gessler *et al.,* 2017). Under a throughfall reduction experiment, we hypothesized that species presenting higher *δ*^13^C due to higher WUE under drought would also present high values of C:N as a result of reduction in nitrogen availability due to drying of the surface soil layer and slower decomposition of litter and/or through disruption of litter fall by the throughfall reduction structure.

Here we analyse the phenological patterns of a tropical rainforest community (337 trees from 30 species) at the Daintree Rainforest Observatory (DRO) in the Wet Tropics of northeastern Australia. We present observations of flowering and fruiting patterns in response to climate in general, as well as to the imposition of a throughfall exclusion experiment. We specifically aimed to characterize (1) the impact of the throughfall reduction on flowering and fruiting activity of the tree community, and (2) how reduced soil moisture affected the stable isotope composition and leaf C:N in four dominant tree species, and to look for and understand any links between physiological acclimation and phenology at the species level as a result of the throughfall exclusion experiment.

## Materials and Methods

### Study site

The phenological observations and physiological measurements for this study were conducted in lowland tropical rainforest at the DRO administered by James Cook University (16°06′20″S 145°26′40″E, 50 m a.s.l.), in northeastern Queensland, Australia. The rainforest at the study site has been classified as complex mesophyll vine forest (Tracey, 1982; Goosem *et al.,* 1999). The vegetation at the DRO has an uneven height and heterogeneous floristic composition, with the canopy comprising a mixture of both mesophyll and notophyll species spread across a wide range of phylogenies (Laidlaw *et al.,* 2007). The canopy is 24–35 m high, with a subcanopy that is a mixture of true shade-tolerant species and shorter sun-tolerant individuals that will eventually become canopy trees. Of the 78 tree species present, 45% occur as upper canopy species. Although there is significant seasonal variation in climate, 95% of all tree species are evergreen. The community also includes lianas and smaller vines, with many of these obtaining canopy height.

**Figure 1:**
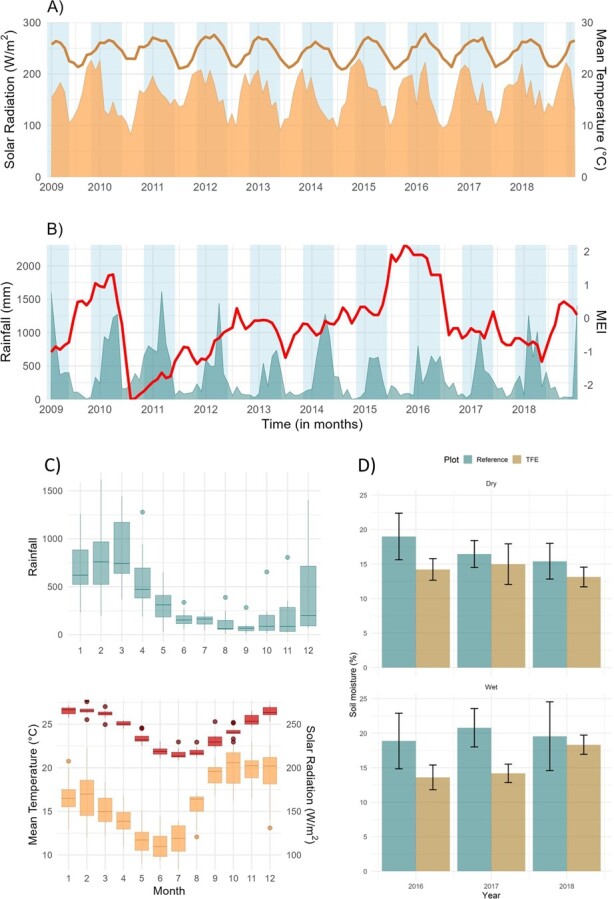
**(**A) Time series of monthly mean temperature (line) and solar radiation (W/m^2^; area); (B) time series of monthly rainfall (mm; shaded area) and Multivariate El Nino Southern Oscillation Index (MEI); (C) seasonal variation in rainfall (blue boxplot), mean temperature (red/darker boxplot) and solar radiation (orange/lighter boxplot); and (D) monthly soil moisture (%) average for all depths in the reference plot and TFE after the commencement of the experiment (2016–2018). Blue background area in (A) and (B) represents wet season.

The Wet Tropics region experiences a seasonal moist tropical climate, with a distinct wet season from November to May and a dry season from June to October (Fig. 1A and C). Minimum rainfall is usually found in September (78.8 ± 78.98 mm) and maximum in March (852.86 ± 371.0 mm). The maximum rainfall of the whole period occurred in February 2011 (1615.6 mm), while the minimum of rainfall from the whole period occurred in September 2014 (4.6 mm). Mean temperature varies from 21.38°C ± 0.58 to 27.02°C ± 0.45, with minimum usually between June and July, with the exception of when it reached the maximum value of 22.94°C in August 2010. Maximum values of mean temperature usually occur between December and February, with the exception of 2017 when it occurred in March (Fig. 1A and C). Solar radiation peaks in the very beginning of the wet season (Fig. 1A), in October (Fig. 1C), and presents minimum values between May and July (Fig. 1C). The region is affected by tropical cyclones (TCs) and ENSO events (El Niño and La Niña). Within the temporal span of our study, the area was slightly affected by TC Ita in 2014 and cyclones that occurred before the start of our phenological observations. Assessing multivariate ENSO index (MEI) data, we determined that the most recent El Niño event occurred in 2015–2016 (moderate), and the most recent La Niña events preceded our observations in 2010–2012 (strong) and in 2018 (weak) (Fig. 1B).

Detailed monthly phenological observations in the 1-ha plot at the DRO (Laidlaw *et al.,* 2007) have been carried out since 2009, with canopy access facilitated by a canopy crane (Stork, 2021). Within this plot, a throughfall reduction experiment (TFE) was established in May 2015, consisting of two rainfall exclusion structures, made of clear-panel roofing, which covered in total 0.4 ha of the study site. The structures were found to reduce soil moisture in upper soil layers by about 30% (Fig. 1D, Tng *et al.,* 2018).

The remaining 0.6 ha of the plot with no treatment provides a baseline reference and as such is referred to as the reference plot. The design of the overall throughfall reduction experiment is a before–after–control–impact (BACI) design (Green, 1979) and throughout we will use ‘before’ (the experiment) to refer to data collected in the reference plot and TFE plots in the years prior to the experiment (period 2009–2012). We will use ‘after’ to refer to data collected in the reference plot and TFE plots in the years after the experiment began (period 2016–2018). The locations of both the reference and TFE plots were chosen so that they would be within the footprint of the canopy crane to enable access to the upper canopy during the experiment.

To assess the effects of the TFE at species level, we selected four tree species: two subcanopy [*Cleistanthus myrianthus* (Hassk.) Kurz and *Myristica globosa* subsp. *muelleri* (Warb.) W.J.de Wilde] and two canopy [*Alstonia scholaris* (L.) R.Br. and *Syzygium graveolens* (F.M. Bailey) Craven and Biffin]. Each species was represented by more than five individuals in both the reference and TFE plots (*A. scholaris n* = 8 in the reference and *n* = 9 in the TFE plot, *C. myrianthus n* = 28 in the reference plot and *n* = 25 in TFE, *M. globosa n* = 23 in the reference plot and *n* = 15 in TFE and *S. graveolens n* = 7 in the reference plot and *n* = 6 in TFE). The selected species are amongst the 10 most dominant species with phenological observations in the plot, with *A. scholaris* comprising 3.3% of the total individuals, *C. myrianthus* comprising 15.7% of the total individuals, *M. globosa* comprising 10.7% of the individuals and *S. graveolens* comprising 3.9% of the total individuals with stem diameter 10 cm or greater at breast height. We tested the effect of the TFE on each of these four species’ flowering and fruiting activity, as well as on the C:N ratio, *δ*^15^N and *δ*^13^C of leaves and *δ*^13^C and *δ*^18^O of wood cellulose.

### Phenological observations

Using the canopy crane, flowering and fruiting activity observations were conducted on a monthly basis. For community analyses, we used the percentage of individuals presenting flowers or fruits and included only the species that had at least four individuals in the whole 1-ha plot. We also selected individual trees that had activity observed at least once and did not die during the whole study period, totalling 337 individual trees (210 in the reference plot and 127 in the TFE) of 30 species (30 in the reference plot and 29 in the TFE). Presence or absence of flowers (flower buds and open flowers) and fruit (unripe and ripe) was recorded monthly from 2009 to 2018. Reproductive phenology for 4 years prior to the implementation of the TFE experiment and 3 years after the implementation of the TFE experiment was utilized for subsequent analyses. Data collected during the years 2013–2015 were excluded, as research station construction and staffing did not permit adequate sampling.

### Meteorological data

Site climate data were collected using a permanently mounted automatic weather station (AWS) and included measurements of rainfall, solar radiation (incoming shortwave) and air temperature. At the study site, the peak mean daily temperature and photoperiod (hours of sunlight per day) occurs in the wet season (from November to May), while peaks in average solar radiation occur towards the end of the dry season (from June to October) (Fig. 1). Across the period January 2009 to April 2014 the AWS used was a Weathermaster 2000 (Environdata; Warwick, QLD, Australia). This unit was replaced in May 2014 with a custom-built weather station, consisting of a data logger (CR1000; Campbell Scientific, Logan, UT, USA), an integrated meteorological sensor (WXT520; Vaisala, Helsinki, Finland), a pyranometer (SP-110; Apogee, Logan, UT, USA) and a tipping bucket rain gauge (RIM8000; Campbell Scientific, Logan, UT, USA).

Beginning in 2014, soil moisture at the site was measured using time domain reflectometry probes (CS616; Campbell Scientific, UK). These were installed at 150-, 100-, 50- and 10-cm depths in eight soil pits, four pits in each of the reference plot and TFE plot. Eight separate data loggers were used to collect soil moisture readings at 15-min intervals. To analyse the soil moisture data for the years of 2010 to 2012, we used a soil pit associated with the site eddy covariance Ozflux station to provide a baseline for regression analyses of sampled soil moisture. Measurements were made at 10-, 75- and 150-cm depths using time domain reflectometry probes (CS615; Campbell Scientific, Logan, UT, USA).

### Stable isotope composition and nutrient analysis

To analyse foliar *δ*^13^C and *δ*^15^N values, and percent carbon and nitrogen by weight from the selected species, we sampled mature leaves (5–10 leaves per tree) from three trees per species in the reference plot and three trees per species in the TFE (*n* = 24 trees, 12 in TFE and 12 in the reference plot). Sampling was conducted in September and November 2018 and February 2019. Sun-exposed leaves were accessed using the canopy crane. The leaves were subsequently dried at 60°C for 7 days and then homogenized using a rock mill grinder (Rocklabs, Auckland, New Zealand).

We also analysed *δ*^13^C and *δ*^18^O values from wood micro-cores sampled at breast height in 2022, in order to capture the treatment period, aiming to capture between 1 and 3 years of wood production based on typical growth rates for the species at the site. Isotope measurements were conducted on three trees per species per treatment that showed flowering and fruiting activity during the study period. Micro-cores were subsequently dried at 60°C for 7 days and had the most recent 2–5 mm of wood sampled. We then extracted cellulose following the Brendel method for small samples (Brendel *et al*., 2000).

Foliar δ^13^C and δ^15^N values, %C and %N by weight were determined using an elemental analyser (ECS 4010 CHNSO Analyser; Costech Analytical Technologies INC, Valencia, CA, USA) fitted with a Costech Zero Blank Autosampler coupled via a ConFloIV to a Thermo Scientific Delta V^PLUS^ using continuous-flow isotope ratio mass spectrometry (EA-IRMS). *δ*^13^C and *δ*^15^N values are reported as per mil (‰) deviations from the Vienna Pee Dee Belemnite (VPDB) and AIR reference scales, respectively. The cellulose extracted from the wood microcores was analysed for *δ*^13^C and *δ*^18^O using a Thermo Scientific temperature conversion elemental analyser coupled via a ConFlo III interface to a Delta V^PLUS^ isotope ratio mass spectrometer. The *δ*^18^O values are reported as per mil (‰) deviations on the Vienna Standard Mean Ocean Water (VSMOW) reference scale. Leaf elemental and isotopic analyses were conducted at the Advanced Analytical Centre, James Cook University in Cairns, Australia, and microcore cellulose isotopic analyses were conducted at the Swiss Federal Institute for Forest, Snow and Landscape Research WSL, Birmensdorf, Switzerland.

### Statistical analyses

#### Effects of the TFE on community phenology

Prior to analysis, any data gaps were filled using Kalman smoothing imputation in the ‘ImputeTS’ package (Moritz and Bartz-Beielstein, 2017). To assess year to year variation in both the reference and TFE plots, we developed generalized linear mixed models [GLMM, family = Poisson(link = ‘identity’)] with proportion of individuals’ flowering and fruiting as the response variables (multiplied by 100 and transformed into integers) and year as the grouping factors for each treatment. To account for repeated observations, we added month as the random effect. We then conducted pairwise comparisons using the marginal means from the fitted models.

To assess if the contrast between the reference and TFE plots differed between the before and after experiment periods, we subtracted the monthly proportion of the reference plot from the TFE plot and used a Wilcoxon rank test to identify significant differences. We then compared before vs after the experiment, for both flowering and fruiting in the community, and for each selected species.

#### Species level

To test the correlation between changes in percentage of flowering and fruiting and changes in soil moisture for each of the selected species, we conducted Spearman correlation analyses between the annual phenological activity and annual soil moisture. Because we wanted to test whether wetter or drier years led to an increase or decrease in phenological activity, we correlated all years from both treatments using the averaged proportion of phenological activity for the years of 2009–2018 in the reference plot and 2016–2018 in the TFE altogether, allowing for plot-specific soil moisture.

To better understand the effects of the TFE on the isotope composition and nutrient concentration of the selected species, and how this is related to possible acclimation to a drier environment, we tested for differences in leaf *δ*^13^C values, *δ*^15^N values and C:N in each season (dry—July, transition—November, and wet—January), and wood cellulose *δ*^13^C and *δ*^18^O values using two-way ANOVA. All analyses were conducted in R (R Core Team, 2019), using the packages ‘timeSeries’ (Wuertz, 2020), ‘glmmTMB’ (Brooks *et al.,* 2017), ‘emmeans’ (Lenth, 2020) and ‘mgcv’ (Wood, 2017).

## Results

### Drought experiment and community phenology

After initiation of the throughfall reduction in the TFE plots, soil moisture decreased on average ~22% in the wet season and ~16% in the dry compared to controls (Fig. 1D). Differences in soil moisture between seasons were less pronounced for the years 2016 and 2018, which is most likely in response to ENSO influences on rainfall patterns (Fig. 1B). Soil moisture averages in the dry season in 2017 and 2018 were lower in the control compared to that in 2016, and the wet season in 2018 had some intense storms resulting in lateral water movement into the TFE, which elevated soil moisture levels (Fig. 1D).

**Figure 2:**
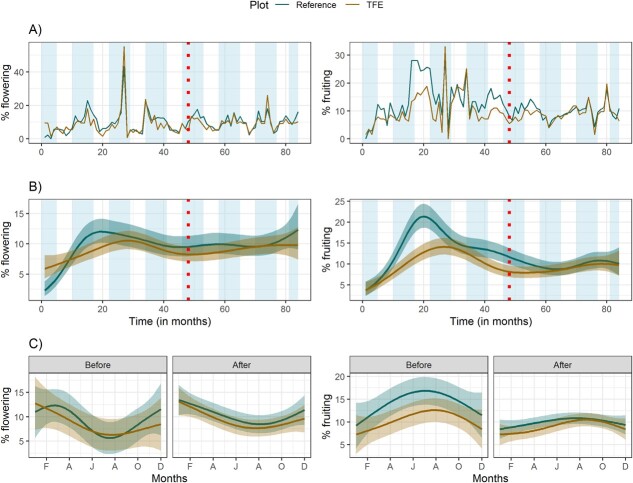
**(**A) Discontinuous time series of actual data of community flowering and fruiting activity in both plots (reference and TFE) from 2009 to 2012 (before) and 2016 to 2018 (after) at the DRO, Cape Tribulation, Australia. Dotted line indicates when the TFE was established in 2016. (B) Fitted generalized additive model responses to the time series of the percentage of individuals’ flowering and fruiting per month since first observation (2009–2018) and (C) fitted generalized additive model responses of intra-annual (seasonality) percentage of individuals’ flowering and fruiting in relation to the month of year. Shades in the curves represent the 95% confidence interval. Wet season duration (shaded bars) indicated in (A) and (B).

**Figure 3:**
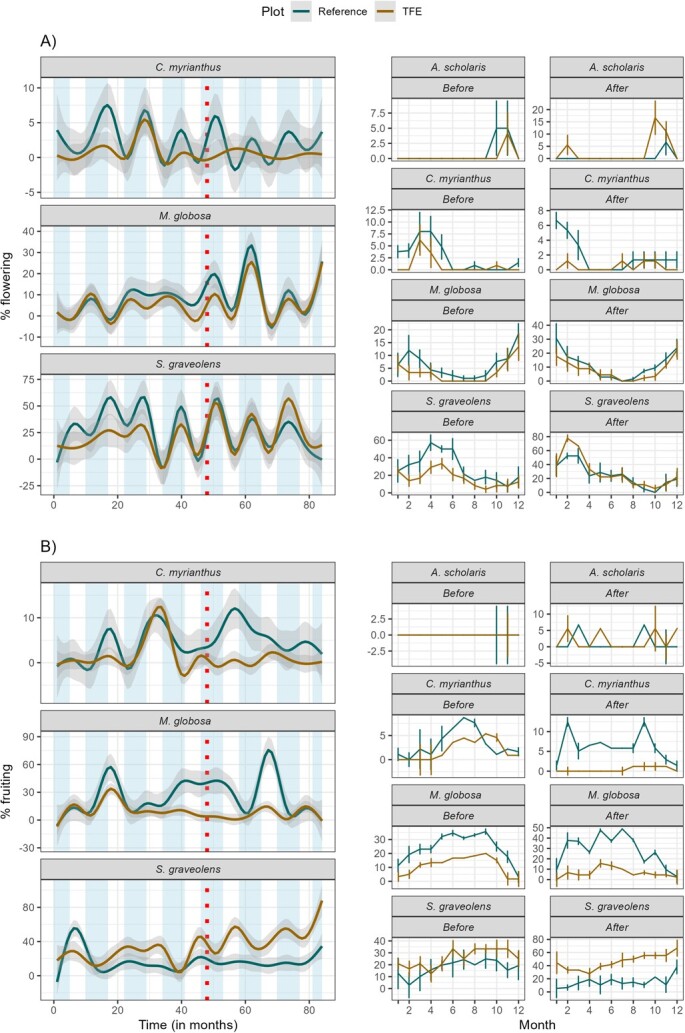
Fitted smoother splines for the time series of proportion of individuals’ flowering (A) and fruiting (B) throughout the study period (left) and in seasonal patterns (right) before and after start of the TFE experiment for the four selected species in both the reference and TFE plots at the DRO, Cape Tribulation, Australia. Wet season duration (shaded bars) indicated in (A) and (B). Dotted line indicates when the TFE was established in 2016.

We observed a significant negative effect of the TFE on the flowering of one species, *S.* g*raveolens*, Wilcoxon test *P* < 0.0001, Table 1), but no overall pattern for the community (Table 1, Fig. 2A and B). With respect to fruiting activity, we observed a significant effect on both the community and two of the four selected species (*S. graveolens* increased and *C. myrianthus* decreased*,*Fig. 3, and Wilcoxon test both *P* < 0.0001, Table 1) due to TFE. This result indicated a relative increase in fruiting activity in the TFE plot after the experiment was initiated, because the difference of reference minus TFE was larger before the treatment and then decreased after treatment (Fig. 2A and B).

**Table 1:** Results from the Wilcoxon rank test for the difference in activity between TFE and reference plot (TFE minus reference) between the periods before and after the experiment initiation for the community and for each selected species. Bold text indicates significant differences.

		Flowering			Fruiting		
		Ratio	*W*	*P*	Ratio	*W*	*P*
Community	Before vs after	0.766	847	0.881	**3.829**	**1260**	**<0.001**
*A. scholaris*	Before vs after	−0.219	974	**0.065**	0	912	0.3361
*C. myrianthus*	Before vs after	1.175	843	0.832	**0.228**	**427.5**	**<0.0001**
*M. globosa*	Before vs after	0.839	845	0.864	0.582	745.5	0.285
*S. graveolens*	Before vs after	**−2.936**	**1316.5**	**<0.0001**	**0.266**	**1348.5**	**<0.0001**

Flowering activity of the tree community (proportion of individuals) at the DRO peaked in the wet season (Fig. 2C), while the peak fruiting occurred in the dry season both before and after setup of the experiment (Fig. 2C). Peak activity values for flowering and fruiting were generally between 25% and 30% of individuals. The highest peaks in flowering activity occurred in both the reference and TFE plots in March 2011, with 43.3% and 55.1% of individuals in activity in each plot, respectively.

### Species level phenology

**Figure 4:**
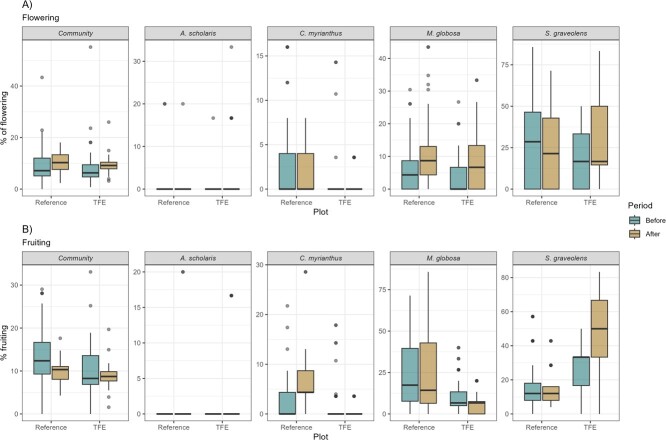
Box plots comparing proportion of flowering and fruiting activity in the periods before vs after start of the TFE experiment at the DRO, Cape Tribulation, Australia, for the community and each selected species.

Phenological patterns differed among the four selected species and they responded differently to the TFE (Table S1). For example, *A. scholaris* experienced a marginally significant (*P =* 0.065) increase in flowering but no significant change in fruiting activity as a result of the TFE (Table 1, Fig. 3). *S. graveolens* experienced a significant increase in flowering and fruiting activity as a result of the TFE (Table 1, Fig. 3), whereas *C. myrianthus* declined in fruiting activity in the TFE. There was no detectable difference in activity observed for *M. globosa* (Table 1, Figs 3 and 4). In the smoothers fitted to the long-term phenological series, it is possible to see that *A. scholaris* presented peaks of flowering activity similarly between the reference and TFE, but after initiation of the TFE, the treated plot presented peaks with high activity (Fig 3A, left). Although fruiting activity of *A. scholaris* was not fully synchronic between reference and TFE, it is possible to see a higher activity in the TFE (Fig. 3B, left). The opposite occurred with *C. myrianthus*, which presented peaks of activity in both reference and TFE in the period before, but no peaks of activity in the TFE after experiment initiation, while the reference plot presented three distinct peaks for flowering and one distinct period of high fruiting activity (Fig. 3A and B, left). *M. globosa* presented similar activity throughout the period between reference and TFE, although after experiment initiation, it is possible to see a peak of fruiting activity in the reference that did not occur in the TFE (Fig. 3B, left). *S. graveolens* presented similar peaks throughout the series, but with flowering activity higher in the reference before and lower in the reference after experiment initiation (Fig. 3A, left). The differences of activity between the plots are visible in the seasonal behaviour of all species (Fig. 3A, B, right).

### Stable isotopes

We observed no significant effect of the TFE on *δ*^13^C_leaf_ across the four species, but there was an effect of season for *C. myrianthus* (two-way ANOVA, *P* < 0.01, Table 3). *S. graveolens* presented a significant increase in *δ*^15^N_leaf_ in the TFE in all seasons (*P* < 0.001, Table 3) and a significant increase in C:N in the wet season compared to the dry season (*P* < 0.03, Table 3). In *C. myrianthus*, leaf C:N was also significantly influenced by the interaction between TFE treatment and season (*P* < 0.05, Table 2).

**Table 2:** Variation in *δ*^13^C and *δ*^18^O from wood microcores at the DRO, Cape Tribulation, Australia, sampled in 2022

Variable	*A. scholaris*		*C. myrianthus*		*M. globosa*		*S. graveolens*		*P*		
	Reference	TFE	Reference	TFE	Reference	TFE	Reference	TFE	Species	Treat	S × T
δ^18^O	27.8 ± 0.1	28.3 ± 0.2	26.9 ± 1.8	28.7 ± 0.4	28.7 ± 0.4	28.7 ± 0.2	29 ± 0.2	28.7 ± 0.3	0.391	0.322	0.464
δ^13^C	−27.5 ± 0.3	−27.1 ± 0.2	−28.8 ± 0.2	−28.2 ± 0.7	−27.7 ± 0.4	−28.8 ± 0.5	−27.2 ± 0.1	−27.7 ± 0.1	**0.009**	0.56	0.108

Values are means ± SEM, *n* = 3 per treatment group. S × T = species × treatment interaction effect.

**Table 3:** Results of two-way ANOVA for each individual species, testing for the differences between TFE and reference plot within each season. Mean values within a row with different superscript letters are significantly different (*p* < 0.05) as analysed using a two-way ANOVA with Tukey post-hoc test.

**Species**	**Variable**	**Reference**				**TFE**				***P***		
		**Dry**	**Trans**	**Wet**		**Dry**	**Trans**	**Wet**		**Treatment**	**Season**	**T × S**
	*δ* ^13^C	−28.9 ± 0.9	−28.3 ± 0.8	−29.1 ± 0.2		−28.7 ± 0.0	−29.4 ± 0.7	−28.8 ± 0.2		0.681	0.941	0.454
	*δ* ^15^N	3.97 ± 0.5	4.03 ± 0.3	4.53 ± 0.3		4.5 ± 0.0	3.93 ± 1.0	5.23 ± 0.7		0.43	0.29	0.76
***A. scholaris***	C	43.6 ± 0.2	44.9 ± 0.2	43.5 ± 1.5		42.4 ± 1.0	42.8 ± 1.0	44.2 ± 0.3		0.25	0.545	0.283
	N	1.97 ± 0.0	2.16 ± 0.1	2.15 ± 0.2		2.08 ± 0.1	1.84 ± 0.3	2.06 ± 0.5		0.6	0.889	0.667
	C:N	22.2 ± 0.6	20.9 ± 1.2	20.4 ± 1.0		20.5 ± 1.1	24.2 ± 3.5	23.9 ± 5.7		0.476	0.905	0.605
												
	*δ* ^13^C	−29.9 ± 0.1^ab^	−29.8 ± 0.3^ab^	−28.8 ± 0.2^ab^		−29.5 ± 0.6^ab^	−30.7 ± 0.3^b^	−28.6 ± 0.8^a^		0.768	**0.01**	0.301
	*δ* ^15^N	4.37 ± 0.4	4.13 ± 0.2	3.97 ± 0.1		4 ± 1.1	3.93 ± 0.9	4.13 ± 0.8		0.819	0.973	0.927
***C. myrianthus***	C	39.3 ± 0.4	40.3 ± 0.6	40.9 ± 0.1		38.4 ± 0.2	41.4 ± 0.5	40.5 ± 1.6		0.912	0.031	0.42
	N	2.21 ± 0.1	2.12 ± 0.1	2.01 ± 0.0		1.87 ± 0.1	1.85 ± 0.1	2.28 ± 0.2		0.237	0.378	**0.034**
	C:N	17.8 ± 1.0^b^	19.2 ± 1.2^ab^	20.3 ± 0.3^ab^		20.6 ± 1.1^ab^	22.4 ± 1.1^a^	17.9 ± 0.8^ab^		0.149	0.182	**0.021**
												
	*δ* ^13^C	−29.9 ± 0.3	−28.5 ± 0.4	−29 ± 0.5		−30.7 ± 0.9	−29.1 ± 0.6	−29.8 ± 0.7		0.153	**0.065**	0.971
	*δ* ^15^N	2.93 ± 0.6	2.7 ± 0.4	2.67 ± 0.6		2.83 ± 0.7	2.43 ± 0.5	2.7 ± 0.4		0.803	0.838	0.961
***M. globosa***	C	40.2 ± 1.1	42 ± 0.7	43.6 ± 0.9		40.3 ± 0.4	42.8 ± 0.5	42 ± 0.4		0.684	**0.01**	0.267
	N	1.75 ± 0.1	1.99 ± 0.1	1.82 ± 0.0		1.62 ± 0.2	1.89 ± 0.1	1.7 ± 0.1		0.247	0.131	0.995
	C:N	23.1 ± 1.1	21.2 ± 1.2	24 ± 0.2		25.8 ± 3.5	22.7 ± 0.4	25 ± 1.9		0.245	0.274	0.885
												
	*δ* ^13^C	−29.3 ± 0.7	−30.6 ± 0.4	−29.9 ± 0.5		−29.5 ± 0.4	−29.7 ± 0.4	−29.6 ± 0.3		0.364	0.245	0.486
	*δ* ^15^N	3.37 ± 0.2^ab^	2.93 ± 0.3^b^	2.8 ± 0.3^b^		4.17 ± 0.3^a^	4.07 ± 0.2^a^	3.47 ± 0.2^ab^		**0.001**	**0.06**	0.612
***S. graveolens***	C	41.2 ± 0.5^b^	43.2 ± 0.5^ab^	43.9 ± 1.0^ab^		41.3 ± 0.1^b^	42.5 ± 0.2^ab^	46 ± 2.0^a^		0.534	**0.007**	0.371
	N	1.47 ± 0.1^ab^	1.5 ± 0.1^ab^	1.53 ± 0.1^ab^		1.71 ± 0.0^a^	1.64 ± 0.0^ab^	1.34 ± 0.0^b^		0.305	0.116	**0.037**
	C:N	28.6 ± 3.0^ab^	28.9 ± 1.8^ab^	28.9 ± 1.4^ab^		24.1 ± 0.4^b^	26 ± 0.4^b^	34.3 ± 2.0^a^		0.655	**0.026**	**0.031**

Values are means ± SEM and significant differences at *P* < 0.1. T × S = treatment × species interaction effect.

There was a significant difference among species in the *δ*^13^C value of wood, with *A. scholaris* having less negative values, but no TFE or seasonal effects (Table 1). The *δ*^18^O_wood_ value was not significantly affected by either species or treatment (Table 1).

## Discussion

### TFE effects on the community phenology

At the community level, our results indicated a small increase in the proportion of trees fruiting in the TFE compared to the reference plot, no change in flowering activity and no major change in the seasonality of reproductive activity. There were different driving environmental conditions ‘before’ and ‘after’ the TFE experiment started, likely triggered by the ENSO switch (seen in the MEI in 2010–2011). Even though the two plots are directly adjacent, fruiting activity in the TFE plot was a little lower than the reference plot before the experiment. After the experiment commenced, fruiting activity in the reference plot dropped while activity in the TFE plot remained broadly the same to become comparable to the reference plot. These results do not support findings from previous studies in which experimental drought has led to a decrease in flowering (Meir *et al.,* 2018). However, Brando *et al.* (2006) also found that throughfall exclusion had no effect on the peak of reproductive activity of *Coussarea racemosa* in the Brazilian Amazon.

As indicated, in the ‘after’ period, the drier environmental conditions were in general less conducive to fruiting as evidenced by the decrease in activity of the reference plot. During this period, the smaller decrease in fruiting activity in the TFE suggests a positive impact of the TFE on fruiting activity under times of environmental stress. However, this result does not mean greater plant fitness, particularly if reduced water availability leads to lower fruit viability, as observed by Brando *et al.* (2006). Increased production of even non-viable fruits may, however, still have several important effects on forest dynamics, as these fruits can serve as resources for fauna species. In addition, the impacts of the TFE may be short-lived if tree species are able to adapt to these new conditions. A long-term TFE in the Amazon, for example, resulted in reduced reproductive output of trees for the first 5 years of the experiment, followed by a recovery of both flowering and fruiting after 10 years (Meir *et al.,* 2018). At the same time, we acknowledge the experimental setup is not perfect, and some trees might get more water available than others due to holes in the experiment structure. We also acknowledge that the lack of responses likely reflects that the contrasts in soil moisture between the control and the exclusion plots were not strong enough to impose a response, which is reflected by the isotope data. In any case, long-term studies are therefore important to assess potential acclimation to new conditions.

However, interpretation of the changes between periods is not straight forward due to the anomalous period in 2010–2011 for both flowering and fruiting, which are related to strong ENSO events that occurred in Australia in those years. The La Niña events in 2010–2012 were two of the most significant ENSO events in Australia’s recorded meteorological history (Australian Bureau of Meteorology, 2012). In the far north of Australia, there was no significant drought, and these were in fact the wettest years of the study.

Increased drought such as what happens in El Niño events has been found to influence flowering activity in Southeast Asia forests (Sakai *et al.,* 2006; Brearley *et al.,* 2007). We have found no evidence of this effect due to throughfall exclusion in this Australian tropical forest. An increase in fruit production in dry years has been suggested to occur due to higher levels of photosynthetically active radiation (PAR) that lead to high photosynthetic activity (Nepstad *et al.,* 2002). This is supported by work of Graham *et al.* (2003) who found an increase in fruit production for two Panamanian tree species when increasing PAR artificially and Chapman *et al.* (2018) who found El Niño events, and increased solar radiation, positively influenced fruiting activity in a forest in Uganda. We have found similar results in this study both at the community and species level where fruiting activity increased. The picture is complex as phenological responses to drought may vary between regions, species and vegetation types. In addition, throughfall reduction experiments only decrease soil moisture, and this differs from natural drought periods as it does not lead to the concurrent effects of lower soil moisture and increased vapour pressure deficit, often coupled with increased temperature and increased solar radiation (Meir *et al.,* 2018).

### TFE effects on species phenology, stable isotopes and nutrients

Phenological responses to the TFE varied significantly among species. Analyses of four focal tree species did not support a consistent community-level response to reduced soil moisture. For example, fruiting in the TFE plot decreased in *C. myrianthus*, increased in *S. graveolens* and did not significantly change in *A. scholaris* or *M. globosa.* Therefore, individual species responded in divergent ways to the experimental drought, and the community as a whole did not show a strong response direction for flowering while exhibiting a slight positive effect for fruiting. A previous study at the same site has shown that a common endemic palm species was significantly affected in both its fruiting phenology and physiology by the TFE (Vogado *et al.,* 2020), which provides additional support for species-specific effects.

Our carbon-stable isotope results for the four focal tree species found that overall there was not a strong treatment effect across leaves and wood. An increase in *δ*^13^C_leaf_ value was expected as drought can affect WUE by changing the ratio of intercellular to ambient CO_2_ concentrations, *c*_i_/*c*_a_, and hence *δ*^13^C_leaf_ value. The *δ*^13^C values suggest that carbon isotope discrimination has not changed consistently across species, and the lack of a significant treatment effect was consistent between leaves and wood. The lack of evidence for altered *δ*^13^C as a result of the TFE is consistent with results found by Pivovaroff *et al*. (2021) at the same site where they explored site water dynamics. This contrasts with a species-specific effect in δ^13^C_leaf_ due to the TFE that was previously reported for an endemic palm species (*Normanbya normanyi*) at the same site, which showed an increase in leaf δ^13^C value (Vogado *et al.,* 2020). These results suggest that the TFE may have led to an increase in WUE in some of the species but not all. When assessing if wood cellulose δ^18^O value, as a proxy for transpiration, was altered in TFE trees as a result of lower soil water availability in shallow soil layers, we found that the four species showed no significant response. The overall isotopic signals from *δ*^13^C_wood_ and *δ*^18^O_wood_ showed no significant effect of the TFE.

The values of the elemental C:N ratio (leaf) did not differ due to the treatment across all four species, but the ratio was significantly higher in the wet season than in the dry season for *S. graveolens* in the TFE, a difference that was not found within the reference plot (Table 2). Leaf loss increased significantly at the end of dry/start of wet season, which was exarcebted by the TFE (data not shown). The seasonal difference in the TFE, therefore, could be explained by the more intense leaf turnover. The lack of significant change in C:N in trees between plots provides no clear evidence of a change in leaf nutrient status as a result of TFE. Sun *et al*. (2020) conducted a meta-analysis of 79 studies across the globe and found an overall decrease in C:N under drought, which was not found in our study. In contrast, Sardans *et al*. (2008) studied the effects of warming and drought on C and N concentrations, allocation and accumulation in a Mediterranean shrubland and found no significant difference for one species under drought, *Globularia alypum*, but an increase in C:N on the other studied species, *Erica multiflora*. These results broadly suggest that responses of C:N to drought seem to be dependent on species and region, and the Daintree TFE data are consistent with this.

To address if rooting depth of the trees increased in the TFE, we measured δ^15^N of the canopy leaves, with the expectation that if trees shifted their water and nutrient foraging to deeper soil layers, they would likely have accessed nitrogen with higher δ^15^N (Craine *et al.,* 2015; Nel *et al.,* 2018; Lorenz *et al.,* 2020). When comparing *δ*^15^N_leaf_ values, *S. graveolens* exhibited higher values under TFE conditions in all seasons, but no change was identified in *A. scholaris*, *C. myrianthus* and *M. globosa.* We interpret this as a species-specific increase in rooting depth caused by TFE in only one species. This indicates that the species has a different capacity to respond and acclimate to drought when compared to the other studied species. At the same study site, Pivovaroff *et al*. (2021) analysed canopy tree responses to reduced soil water availability by quantifying changes in plant hydraulic and carbon traits, and concluded that the TFE led trees to uptake water from deeper soil layers, which was also variable by species.

Both phenological and physiological responses to TFE were variable among species. *S. graveolens* presented an increase in both flowering and fruiting activity during the TFE. Flowering activity also increased in *A. scholaris*, while *C. myrianthus* and *M. globosa* exhibited reduced or equal flowering and fruiting activity. Apgaua *et al.* (2015) previously found that *S. graveolens* had traits conducive to drought resilience, such as high sap velocity and rate of sap flow, and moderate WUEi, contributing to an extremely low vulnerability index (based on wood vessel packing per unit area), while *M. globosa* had a higher drought vulnerability. The effects of the TFE on the physiological proxies for drought resilience such as *δ*^15^N values show that *S. graveolens* may be more resilient to drought, with evidence of increased WUE and increased root access to deeper layers in the soil. These plastic responses in species physiology would allow for increased carbon uptake, and this is consistent with the increase in fruiting activity observed for this species in the TFE. On the other hand, *A. scholaris*, *C. myrianthus* and *M. globosa* did not show increased water and carbon uptake, which was reflected in unchanged or decreased phenological activity.

Both phenological and physiological data indicate species-specific responses to drought, as found previously for temperate species (Ogaya and Peñuelas, 2007). This suggests that in this lowland rainforest, the responses to climate change will be heterogeneous, and a hotter, drier climate is likely to affect some tree species more than others and, through this, alter forest floristic composition. Such changes in floristics may not be detected when analysing phenological changes at the community level as changes at the community level may be clouded by species diversity and divergent species responses. An understanding of species level phenology and physiological response to environmental drivers will be required to assess and predict the future impacts of climate change on forest composition and function.

## Conclusion

The overall community-level phenology response to throughfall reduction in the absence of a change in temperature or Vapour Pressure Deficit (VPD) was likely buffered by the high species diversity and lack of general responses among species. In natural drought, there is typically an increase in the length of the period with elevated temperature and VPD (Meir *et al.,* 2018) as well as a decrease in soil moisture. Experimental manipulations of soil moisture, such as TFE, assess only one aspect of a drying climate, and this is a significant limitation when using TFE experiments to study drought effects on tropical tree communities. Additionally, the species level changes in phenology were influenced by physiological processes, which in turn are dependent on functional traits. These results suggest that, in highly diverse tropical rainforests, species may respond very differently in the face of climate change, making predictive modelling of community responses particularly challenging. More detailed studies relating changes in phenology to changes in physiological processes and species-specific traits are clearly necessary to better understand how species and communities will respond to predicted and ongoing changes in climate.

## Supplementary Material

Web_Material_coad064

## Data Availability

The data underlying this article will be shared on reasonable request to the corresponding author.
