# Supplementary material for: Assessing the effects of a drought experiment on the reproductive phenology and ecophysiology of a wet tropical rainforest community
Source: Conserv Physiol. 2023 Sep 19;11(1):coad064. doi: 10.1093/conphys/coad064 (PMC10509008; doi:10.1093/conphys/coad064)
Supplement: Web_Material_coad064 [file web_material_coad064.zip › Supplementary.pdf]

Supplementary information

Table S1. Pairwise comparison of marginal means from the GLMMs of reproductive activity in relation to month for all years in the reference plot and TFE.

	Reference plot								TFE							
		2009	2010	2011	2012	2016	2017	2018		2009	2010	2011	2012	2016	2017	2018
Flowering	2009								2009							
	2010	**-4.936**							2010	-1.07						
	2011	**-5.454**	-0.75						2011	**-4.88**	**-3.90**					
	2012	-3.074	1.968	2.617					2012	-0.31	0.75	**4.59**				
	2016	**-4.318**	0.623	1.332	-1.329				2016	-2.05	-0.99	2.95	-1.736			
	2017	**-4.318**	0.623	1.332	-1.329	0			2017	-2.38	-1.33	2.62	-2.069	-0.338		
	2018	**-4.663**	0.238	0.964	-1.703	-0.378	-0.378		2018	-3.01	-1.97	1.98	-2.707	-0.99	-0.653	
		2009	2010	2011	2012	2016	2017	2018		2009	2010	2011	2012	2016	2017	2018
Fruiting	2009								2009							
	2010	**-8.213**							2010	**-4.98**						
	2011	**-5.299**	3.141						2011	**-6.37**	-1.56					
	2012	**-4.172**	4.475	1.266					2012	-2.28	2.85	**4.35**				
	2016	-2.449	**6.147**	3.011	1.789				2016	-2.28	2.85	**4.35**	0			
	2017	-1.147	**7.293**	4.258	3.081	1.317			2017	-2.34	2.78	**4.28**	-0.07	-0.07		
	2018	-2.572	**6.034**	2.89	1.665	-0.126	-1.442		2018	**-3.44**	1.66	3.19	-1.21	-1.21	-1.14	
